# Report from the World Health Organization’s immunization and vaccines related implementation research advisory committee (IVIR-AC) meeting, Geneva, 11–13 September 2023

**DOI:** 10.1016/j.vaccine.2024.02.014

**Published:** 2024-03-07

**Authors:** Philipp Lambach, Walt Orenstein, Sheetal Silal, Alyssa N. Sbarra, Mitsuki Koh, Rakesh Aggarwal, Habib Hasan Farooqui, Stefan Flasche, Alexandra Hogan, Sun-Young Kim, Julie Leask, Paula M. Luz, Dafrossa C. Lyimo, William J. Moss, Virginia E. Pitzer, Xian-Yi Wang, Joseph Wu

**Affiliations:** a Immunizations, Vaccines and Biologicals, World Health Organization, Geneva, Switzerland; b Emory University, Atlanta, United States; c Modelling and Simulation Hub, Africa, University of Cape Town, Cape Town, South Africa; d Centre for Global Health, Nuffield Department of Medicine, Oxford University, Oxford, United Kingdom; e Department of Infectious Disease Epidemiology, London School of Hygiene & Tropical Medicine, London, United Kingdom; f Jawaharlal Institute of Postgraduate Medical Education & Research, Puducherry, India; g College of Medicine, QU Health, Qatar University, Qatar; h Centre for Mathematical Modelling of Infectious Diseases, London School of Hygiene & Tropical Medicine, London, United Kingdom; i University of New South Wales, Sydney, Australia; j Seoul National University, Seoul, South Korea; k School of Public Health, University of Sydney Sydney, Australia; l Instituto Nacional de Infectologia Evandro Chagas, Fundação Oswaldo Cruz, Rio de Janeiro, Brazil; m Consultant, Tanzania; n Johns Hopkins Bloomberg School of Public Health, Baltimore, MD, United States; o Yale School of Public Health, New Haven, CT, United States; p Institutes of Biomedical Sciences, Fudan University, Shanghai, China; q School of Public Health, The University of Hong Kong, Hong Kong

## Abstract

Evaluating vaccine-related research is critical to maximize the potential of vaccination programmes. The WHO Immunization and Vaccine-related Implementation Research Advisory Committee (IVIR-AC) provides an independent review of research that estimates the performance, impact and value of vaccines, with a particular focus on transmission and economic modelling. On 11–13 September 2023, IVIR-AC was convened for a bi-annual meeting where the committee reviewed research and presentations across eight different sessions. This report summarizes the background information, proceedings and recommendations from that meeting. Sessions ranged in topic from timing of measles supplementary immunization activities, analyses of conditions necessary to meet measles elimination in the South-East Asia region, translating modelled evidence into policy, a risk-benefit analysis of dengue vaccine, COVID-19 scenario modelling in the African region, therapeutic vaccination against human papilloma virus, the Vaccine Impact Modelling Consortium, and the Immunization Agenda 2030 vaccine impact estimates.

## 1 Context

Vaccination policies should be informed by rigorous evidence. A vital aspect of understanding the potential value of vaccines is predicting their impact on future infectious disease burden. As an advisory group to WHO, the Immunization and Vaccine-related Related Implementation Research Advisory Committee (IVIR-AC) reviews and provides feedback on various vaccine value assessments, effectiveness and impact studies, and modelling analyses of specific priority to the Strategic Advisory Group of Experts on Immunization (SAGE) and the Immunization, Vaccines, and Biologicals (IVB) Department.

The main role of IVIR-AC is to provide advice and recommendations to WHO. It has no executive or decision-making power [1]. IVIR-AC holds bi-annual meetings. This report summarizes proceedings and recommendations of the IVIR-AC meeting held from 11 to 13 September 2023 in Geneva [2].

## 2 Scope and objectives of meeting

Eight meeting sessions covered on various topics set as priorities by WHO IVB and SAGE including: modelling of the impact on measles incidence of timeliness versus coverage achieved during measles supplementary immunization activities (SIA; i.e., mass immunization campaigns); modelling on measles/rubella elimination in South-East Asia; translating vaccine impact modelling into immunization strategy, policy and programme decisions; benefit-risk assessment: dengue vaccine; mathematical modelling of the COVID-19 pandemic according to different vaccination scenarios in Burkina Faso and Cameroon; therapeutic HPV vaccine impact modelling; Vaccine Impact Modelling Consortium; and Immunization Agenda 2030 vaccine impact estimates. Specific objectives and questions posed to IVIR-AC are described for each session below.

## 3 Description of sessions

### 3.1 Session 1: Modelling of timeliness versus coverage of measles supplementary immunization activities

General guidance suggests that population-level immunity for measles must be greater than 95 % in all subnational units, age groups and population subgroups to achieve elimination, which requires very high levels of immunization coverage of measles containing vaccine (MCV). As with all vaccine programmes, first-dose MCV (MCV1) coverage faced COVID-19 pandemic-related disruptions. However, MCV coverage recovery compared to coverage from other vaccines has been slower to rebound, particularly in low-income countries. Additionally, in recent years, there have been an increasing number of large or disruptive measles outbreaks. WHO recommends that countries continue supplementary immunization activities (SIAs) with MCV to maintain population immunity until routine coverage reaches 90–95 % for both MCV1 and MCV2 coverage. To prevent measles outbreaks, follow-up SIA campaigns are currently recommended to be conducted before the number of susceptible children reaches the size of an annual birth cohort and should prioritize reaching previously un- and under-vaccinated children.

SIAs are currently planned based on the accumulation of susceptible children, which in practice is approximated using routine vaccination coverage. For example, countries where routine MCV1 coverage is less than 60 % are recommended to conduct yearly SIAs. As measles is highly seasonal, it is known that the impact of SIAs can be substantially increased by their implementation before peak transmission season, which suggests that if SIAs are delayed there may be large implications for preventing infections. WHO partners have increasingly focused on achieving high quality campaign implementation in recent years, but the criteria used to define ‘quality’ have prioritized high coverage at the expense of timeliness (i.e., the need to conduct a campaign prior to anticipated measles outbreaks). Technical advice, based on expertise and experience, has been consistent to not delay SIAs, yet programmatic and funding decisions have had a major impact on measles control in some countries with outbreaks starting before campaigns were implemented.

During the session, the Institute for Disease Modeling (IDM) presented results from an analysis of the relationship between timeliness and coverage of SIAs in preventing measles outbreaks and determining campaign quality. Given that delays to SIAs occur (e.g., in the last decade, there have been six outbreaks in the African Region that occurred between the period an SIA was planned and when it was ultimately implemented), the IDM team tried to quantify the relative benefit of having a lower coverage campaign that was implemented “on-time” (i.e., 4 months prior to outbreak) versus campaigns with higher coverage that were delayed. The team used Epidemiological MODeling software [3] (EMOD) to run stochastic simulations of measles outbreaks from a generalized population with assumed hypothetical fixed routine vaccination coverage of both 30 % and 70 % and showed that outbreaks occurred with varying periodicity and severity. Planned SIAs were generated to occur using a fixed set of simulation trajectories with respect to birth rates, seasonality, case importation and routine immunization coverage in an iterative process. After identifying an outbreak in a simulation, an SIA (that conservatively assumed doses would first be administered to previously vaccinated children) would be planned four months prior to the start of an outbreak, and the simulation was re-run. The team ran various hypothetical scenarios with campaigns delayed up 6 months (i.e., 1, 2, 4 and 6 months) and with varying coverage levels (i.e., 50 %, 70 %, and 95 %).

Generally, the team suggested that their findings indicate that delayed campaigns decrease impact by reducing the number of infections averted per dose. Additionally, in a low routine immunization context (e.g., 30 % routine coverage), scenarios with on-time SIAs (e.g., 1-month delay) with lower coverage (e.g., 50 %) yielded fewer infections than SIAs delayed by 6 months (i.e., that occurred 2 months after the outbreak) that reached high coverage (e.g., 95 %). In settings with higher routine immunization coverage (e.g., 70 %), campaigns needed to both be timely and reach high coverage in order to modify the size outbreaks. Results also indicated that in SIAs where doses were given to all children uniformly (i.e., persons who are unvaccinated and vaccinated are equally likely to be vaccinated), lower coverage on-time campaigns (i.e., 4 months prior to outbreak) are more impactful than delayed campaigns with high coverage. The team additionally noted that as SIA coverage increased, so did the time interval until the next outbreak.

To summarize various ongoing workstreams and interests in overall measles SIA modelling, an additional presentation was made by the London School of Hygiene & Tropical Medicine representing a VIMC Measles SIA Working Group. Five priority areas were identified to be of interest to modelling groups and policy, funder and programme stakeholders, which included:

• Investigating factors related to transitioning away from SIAs (e.g., outbreak risk, impact of new technologies such as delivery tools, use of school-based vaccination checks);
• Exploring how to best adapt current SIA strategies at subnational, national and regional scales;
• Evaluating SIA strategies to be used for outbreak response, such as modelling the effectiveness and cost-effectiveness of vaccinating only outbreak-affected subnational units versus a wider geographic range;
• Quantifying and interpreting immunity gaps in relation to the effectiveness of SIAs; and
• Understanding the implications of SIAs for measles and rubella elimination, such as what can be learned from rubella elimination and its implications for measles elimination.

IVIR-AC was asked to provide feedback on the robustness of the methods used by IDM to justify generalized conclusions regarding the balance of timeliness and coverage in determining SIA quality and their overall utility in programmatic decision making.

IVIR-AC feedback and recommendations

IVIR-AC agreed that the methods presented by IDM are appropriate for illustrating that high-quality SIAs are a function of both timeliness and coverage, though further contextualisation is required before the modelling methods and results can be applied to specific settings. The committee notes that in general, achieving high coverage campaigns while allowing for adequate planning horizons is critical; nevertheless, if an outbreak is imminent, nominal reductions in coverage in SIAs deployed prior to the outbreak could prevent more infections compared to delaying the campaign. IVIR-AC recommends the following:

• While the model results are valuable for general insights, they are not appropriate for guiding the timing of SIAs in specific settings; for specific settings, it is necessary to account for immunity gaps on national and subnational scales, as well as uncertainty in the size and timing of outbreaks geographically.
• It is difficult to know when measles outbreaks will occur, and long planning horizons may be needed to implement SIAs. To allow the modelling results to be interpreted in light of different planning horizons, the results should also be presented in terms of how many months before the outbreak, the SIA is implemented (e.g., −3, −1, 0, 2 months), rather than the delay from the proposed planning time.
• Model results should be presented for multiple, longer time periods, rather than for only 1 year, as low coverage campaigns may only slightly delay measles outbreaks. At a minimum, a 2-year time periods should be used to represent settings where SIAs are planned to occur every 2 years.
• For future work, it is important to understand the barriers to high immunization coverage through routine systems and the extent to which these barriers are or are not overcome through SIAs compared with other strategies and technologies (e.g., microarray patches, rapid diagnostic tests for tailoring where services are delivered) to complement modelling the impact of SIAs.
• The conservative assumption that initial SIA doses are administered to those who were previously vaccinated somewhat limits the range of parameter space to explore. A scenario in which SIA doses are targeted to those who did not receive previous vaccination should also be modelled.
• The development of new analytic tools is needed to identify immunity gaps and to better predict when outbreaks might occur. It is also important to understand the extent to which seroprevalence studies and other data are representative of the target population for SIAs (i.e., unvaccinated persons) when assessing the value of such data for informing the timing and the geographic tailoring of SIAs.

Additional recommendations include:

• For the appropriate contextualization of modelling results, the coverage assumptions in the model for routine immunization and SIAs should be based in actual data. Additionally, coverage estimates and the duration of campaigns from various countries should be presented in a supplementary table.
• IVIR-AC recommends referring to the “efficiency” (or “impact ratio”) of the vaccination programmes when presenting results, referring to infections averted per 100 doses, since there was no formal cost-effectiveness analysis conducted.

### 3.2 Session 2: Modelling on measles/rubella elimination in South-East Asia

In 2010, an expert advisory panel convened by WHO deemed that measles theoretically can and should be eradicated, and these findings were endorsed by SAGE in 2011. SAGE also noted that necessary conditions for a global effort have not been met and the focus should be on making progress toward those conditions prior to assessing the feasibility of an eradication effort. Since then, measles and rubella elimination targets have been set by the Regional Committees of all WHO regions. Committing to elimination is critical for strategically allocating necessary resources and rallying political will at regional and country levels. The Feasibility Assessment of Measles and Rubella Eradication presented to SAGE in 2019 established that setting a global eradication goal would not be considered until establishing two key elements: further progress on reaching regional elimination targets, and the development of agreed scenarios of epidemiology, technology, and financing from which a final push towards eradication would be feasible and warrant a final push and investment. Furthermore, measles and rubella vaccination programme targets and timelines for achieving elimination have been seriously disrupted due to the COVID-19 pandemic, calling for an increased focus on strategic planning, necessitating setting evidence-based goals, and action toward those goals.

In order to achieve regional measles or rubella elimination, the following criteria must be met by each country in the region:

• Elimination is verified at the national level, which must be confirmed by a Regional Verification Commission
• Endemic lineages (i.e., chains of transmission) are interrupted for more than 12 months and verified for more than 36 months. This does not include circulation for less than 12 months of imported lineages.
• High-quality, laboratory supported surveillance systems with adequate sensitivity and specificity are required to detect, notify, and investigate suspected cases and outbreaks in a timely manner and classify cases by source.

As of August 2023, 83 countries (but no regions) had verified measles elimination status; for rubella, 98 countries (2 regions) had achieved elimination status.

Combining modelling and operational experience can be an effective tool for developing objective, evidence-based reviews of timelines and goals. A VIMC project working group with representatives from the University of Georgia and Pennsylvania State University presented an overview of scenario modelling work [4] previously performed of the countries in WHO’s South-East Asia Region. The project was framed to explore the feasibility of reaching the conditions necessary for achieving elimination under various projection scenarios, rather than estimating the probability that elimination would actually occur. The overall approach was to develop possible vaccination scenarios, to run simulations to generate outputs, present findings to WHO Regions, and hold country consultations to share results. There were two models used for both measles and rubella by the VIMC working group which each had different model structures, assumptions on seasonality, vaccine effectiveness and population mixing, and methods for including case importations. Both rubella models concluded that there was a high probability of reaching the conditions necessary for achieving rubella elimination in the South-East Asia Region by 2025 under all vaccination scenarios, and both measles models suggest a high probability of achieving the conditions necessary for measles elimination in the Region by 2030 under all considered vaccination scenarios. Specific scenarios across the different measles and rubella models suggest slightly shorter timelines were possible for achieving conditions necessary for elimination however these scenarios assume optimal conditions of vaccine coverage improvement, population mixing, and seasonality.

Generally, the results generated by the VIMC project team were well received and were seen as a useful starting place to guide discussions, although it was unclear how these results will be used to inform programmatic decisions at the time the project concluded. During the session, the project team discussed lessons learned during the process of completing the project, which mainly included incorporating country programme staff in the process of generating coverage scenarios. The project team also discussed the feasibility of adapting this approach for other regions.

IVIR-AC was asked to provide feedback on how the methodology can be strengthened and comment on the utility and generalizability of the methods for adaptation in other regions.

IVIR-AC feedback and recommendations

IVIR-AC agrees with the overall approach and concept of using multiple models and agreed-upon feasible vaccination scenarios to understand which scenarios would be most effective for achieving elimination in specific countries. IVIR-AC agrees that these scenario-based analyses could be extremely useful for developing realistic elimination goals. Further consideration should be given to how to adapt the models to be suitable to other settings as scenarios to estimate the potential of reaching conditions necessary for elimination in SEAR are likely to be useful to other regions. Additional recommendations include the following:

• IVIR-AC recommends updating the vaccination scenarios while considering the impact of the COVID-19 pandemic and potential immunity gaps in adolescents and adults.
• When considering applying this framework to other regions, it would be crucial to understand what elements of model design and parameterisation can be readily adapted to the other regions (e.g., population demography, historical vaccine coverage, historical transmission assumptions) and what elements would remain unchanged (e.g., assumptions about disease characteristics), while ensuring that model scenarios are realistic and country-specific.
  o IVIR-AC recommends providing more details on each of the vaccination scenarios, especially regarding the age groups targeted by SIAs, since the five vaccine scenarios used for countries in the South-East Asia Region might not be the most appropriate scenarios for other regions.
• For the elimination of measles and rubella, issues related to operational feasibility often also pose a great challenge. Of these, failure of service delivery to fill immunity gaps, insensitive surveillance, and vaccine refusal are extremely critical. Modellers should consider incorporating additional model parameters to account for the sensitivity of surveillance systems to strengthen the transferability of models.

Additional recommendations include:

• IVIR-AC recommends unifying the terminology used across presented reports. The publication [4] has many outcomes (e.g., elimination threshold, timing of achieving the threshold, duration of continuous-time periods spent below the threshold) and in contrast, the SEAR report focused on the probability of elimination (i.e., of having an annual incidence of 5 infections per one million people or less) for a given year and also the mean probability of achieving conditions required for elimination in each year.
• IVIR-AC agrees that the threshold based on 5 infections per million persons or less was used only for the purposes of the modelling and not for the purposes of surveillance (where the definition is still interruption of endemic lineages for more than 12 months), which was meant to be conservative. It would be useful, however, to have more information on how that threshold was derived (e.g., if it is based on maintaining chains of transmission or an equivalent measure).
• IVIR-AC recommends integrating the results of the two models for both measles and rubella into one graph and comparing the uncertainty of the results between models.

### 3.3 Session 3: Translating vaccine impact modelling into immunization strategy, policy and programme decisions

Modelling has played an increasingly important role in informing immunization strategy, policy, and programme decisions including at WHO. Priority modelling-related activities in IVB focus include coordination of modelled estimates to inform IA2030 and other global level strategies, convening experts to review and advise on methods and results to inform global policy (e.g., IVIR-AC), and collaborating with country, regional and global partners to increase capacity for using and generating modelled evidence. Many countries, however, have not yet systematically incorporated modelled evidence into their immunization strategy, policy and programme decision-making process, often because of capacity constraints.

Modelling has contributed to an increasing role in informing immunization strategy, policy and programmatic decision making. However, there is a lack of tools to assess the quality of available models and modelled evidence and often interpreting, translating, and implementing modelled evidence requires a high level of expertise or training. To support the appropriate use of modelling evidence in countries, the WHO Secretariat and IVIR-AC have established a sub-group [5] that will oversee the development of guidance by 2025 on translating vaccine impact modelling into strategy, policy, and programme decisions for immunization. Desired outcomes of this project include:

• Modellers to incorporate best practices for modelling and adapt communication to better inform countries, and provide decision makers with approaches on how to effectively use modelled evidence;
• Country decision makers to effectively use modelled evidence to inform strategy, policy, and programme decisions in the immunization field; and
• Both decision makers and modellers to implement and maintain impactful collaboration to inform decisions.

During the session, the working group chair presented an overview of the commission and rationale for the subgroup. Activities of the subgroup in 2023 include drafting proposed components of the guidance document, holding a regional meeting to receive input from WHO Regional Offices on ongoing and planned modelling initiatives, and conducting a needs assessment. Potential components of the guidance document include how decision makers can effectively use modelled evidence (e.g., multi-model comparison, understanding uncertainty), how modellers can effectively conduct analyses and communicate results to inform decisions (e.g., clarifying model limitations, tools for disseminating results), and how decision makers and modellers can better collaborate (e.g., formulating questions, developing scenarios). Key findings from the regional meeting include the following:

• Modelling should be integrated into routine practice and should be intuitive, user-friendly, and regularly updated;
• Modellers should directly engage with Regional Immunization Technical Advisory Groups (RITAGs), National Immunization Technical Advisory Groups (NITAGs) and National Immunization Programs to identify modelling questions, and build capacity to generate and use modelling within countries, by co-developing modelling questions, collaborating on collecting, reviewing and using data, and discussing how results can be applied to policies; and
• As modelling is not commonly used by NITAGs in most LMICs in the African region, it will be important to increase the capacity of NITAGs to digest and critically appraise the evidence.

By 2024, the documentation from the perspective of modellers and decision makers will be developed to guide the effective implementation of approaches for modelling-decision translation. Following in 2025, the subgroup will tailor the guidance document to the needs of various decision-making groups at global, regional, and country scales. For example, this guidance will be tailored to the needs of NITAGs and immunization decision makers and take the form of a document, training modules, or other desired formats.

A working group member reported on plans for the needs assessment which is comprised of a qualitative study interviewing users of modelled evidence, which aims to identify how modelling is used by decision makers currently, the needs and challenges faced by users of modelled evidence, and the types of guidance that will be most useful to users of modelling results, to ultimately inform the content and effective delivery of the guidance. Over 20 key informants, from various WHO regions and country income levels, global and regional agencies, and representing various decision categories (e.g., direct users of modelled evidence such as NITAG members, stakeholders who make immunization programme decisions such as EPI managers), will be interviewed over Zoom or telephone in English, French, Spanish or Russian. Results from interviews will be transcribed and coded using an inductive and deductive framework analysis to examine how modelling is currently being used, needs and challenges encountered, forms of guidance that would be most useful, and other identified themes (e.g., variation in understanding of modelling). During the session, a revised use case statement of mathematical modelling specific to the guidance was defined based on initial interview feedback and was proposed to be:

*“Mathematical models are used to develop scenarios on how vaccination might help to prevent disease in a population compared to the current situation.”*

IVIR-AC was requested to review the presentation, provide feedback, and seek clarification on content, specifically regarding the ability of the planned activities to achieve the aims of the subgroup and on the plans for the qualitative needs-assessment study.

IVIR-AC feedback and recommendations

Following their support of this subgroup and review of the presentation, IVIR-AC recommended that the guidance documents should be conversant with and complement existing WHO resources for policy makers, and should specifically aim to elucidate model complexity, capabilities, and limitations. Additional recommendations include the following:

• To reformulate the definition of modelling to be specific to the role of modelling in immunization and distinguished from other types of modelling (e.g., economic, statistical).
• To increase the relevance of the guidance, the content of the guidance should discuss problem formulation, data contextualization, factors affecting generalizability and uncertainty of modelling results, and acknowledge limitations and assumptions.
• To demystify model complexity, the guidance should provide simple definitions with explanations of common modelling concepts, including highlighting the importance of knowledge exchange.
• To exemplify the guidance delivered, examples could be used, in addition to historical use cases.
• To reduce the risk of inappropriate use of modelled evidence, the guidance should be clear on limitations, including but not limited to model structure, inherent uncertainties in the modelling results due to erroneous models or assumptions, biological systems that are not fully understood, and a need for reliable local data for parameters that are or can be influential to model results.

### 3.4 Session 4: Benefit-risk assessment: dengue vaccine

As incidence continues to rise, dengue is a growing global concern with most cases observed in the Americas, South-East Asia and Western Pacific Regions [6], [7], [8]. To combat the rising burden of dengue, Takeda has developed a tetravalent dengue vaccine candidate (TAK-003) which is based on a live-attenuated dengue serotype 2 virus (DENV2) backbone and designed to provide protection against all 4 dengue serotypes. TAK-003 has recently successfully completed Phase III clinical trials [9], has been licensed in Indonesia [10], and has received approval for use by the European Medicines Agency [11]. However, overall attack rates in the vaccine trial were lower among both placebo and vaccine recipients for DENV3 and DENV4 and in years 4 and 5 [9]. Given the limited power and the experience of the previous live-attenuated tetravalent Dengvaxia vaccine, the trial data was unable to provide evidence against a theoretical, but biologically plausible, safety concern of enhanced DENV3 and DENV 4 disease among seronegative vaccinees.

In order to provide preliminary guidance and to inform SAGE’s evidence review process for TAK-003, IVIR-AC and SAGE Secretariats jointly launched an open call for teams to model the population benefit and individual level risk for TAK-003. Specifically, teams were asked to provide evidence on the following questions:

(1) What are the estimates of population-level and individual-level benefit/risk over 10 and 20 years, stratified by age of recipient, serostatus of recipient and by average transmission intensity in a setting?
(2) What is the cost-benefit of vaccination programmes without pre-vaccination screening for serostatus, or by pre-vaccination screening dependent upon seroprevalence in a specific age group (e.g., pre-vaccination screening in low seroprevalence settings, and no pre-vaccination screening in high seroprevalence settings)?
(3) What is the threshold seroprevalence for pre-vaccination screening by when such an effort becomes either cost-effective or has the most favourable benefit-risk ratio?

To support recommendations at the SAGE meeting on 25 September 2023, IVIR-AC reviewed work of the two selected modelling teams (Imperial College London and University of Notre Dame) during the session.

The team from Imperial College London presented methods and results from a series of models to estimate both individual- and population-level risks and benefits of the introduction of TAK-003. The team first designed an antibody decay model to estimate vaccine efficacy over time by serotype and serostatus that was calibrated to data observed from the clinical trial. This method links antibody titre information with risk ratios of symptomatic disease and hospitalization in a modified version of a correlate of protection model [12]. The team then constructed a stochastic four serotype dengue transmission model updated from a previous deterministic version [13]. The model is age- and serostatus-specific, assumes permanent and complete immunity following infection with homologous serotypes and temporary cross-protection against heterologous serotypes, and incorporates information on time-varying, serotype-specific vaccine-induced protection as described above. The team used multiple models with various combinations of possible vaccine’s mechanism of action (e.g., against disease or infection, and waning vaccine efficacy) and simulated the impact of routine vaccination across settings with different transmission intensities over 5-, 10- and 20-year time horizons for scenarios with and without pre-vaccination screening. The team estimated vaccine impact at the population-level by calculating the proportion of cases averted in the entire population and at the individual-level by calculating the absolute number or proportion of cases averted in the first vaccinated cohort.

The team from the University of Notre Dame also presented methods and results from models used to estimate the population-level impact of TAK-003 introduction. The team designed a transmission model that tracks vaccination status and the number of previous infections without tracking specific serotypes. The model uses environmentally driven parameters to account for seasonal transmission and is calibrated to account for variation in the force of infection across approximately 1800 cities globally with populations greater than 100,000 persons. Model parameters regarding vaccination depend on serostatus and represent a range of serotype-specific efficacy based on an analysis of the TAK-003 clinical trial data. Models were run for a 10-year time horizon, where each realization of the model is for a different city with varying dengue epidemiology. Various model comparisons were made, including across different levels of vaccine coverage, both with and without pre-vaccination screening and various sensitivity and specificities of screening tests, various levels of infectiousness of asymptomatic cases, the underlying mix of serotypes, and the extent to which the vaccine could block infections. Models were additionally run with vaccine parameterization based on features of the previous Dengvaxia vaccine and results were compared to those using TAK-003 parameterization. Population-level impact was assessed by computing the number of cases averted across the entire population.

IVIR-AC was asked to assist in assessing the presented evidence and provide feedback on the appropriateness of the modelling approaches and assumptions, and the robustness of the results.

IVIR-AC feedback and recommendations

IVIR-AC concluded that the modelling results from both groups were generally consistent in suggesting an overall positive population-level impact of TAK-003, which was greater in higher transmission settings. The models suggested that under certain circumstances, seronegative vaccinees could have an increased risk for DENV3 and/or DENV4 disease. In light of limited evidence, further studies are needed to better understand the nature of the vaccine protection against all serotypes. Additional recommendations include the following:

• Despite differences in their approach for interpreting trial results, both models are appropriate for evaluating the potential impact of TAK-003 if communicated alongside their respective assumptions.
• While pre-vaccination screening to identify previous infection could minimize the risk of enhanced disease at the individual level, its use is predicted to considerably reduce the population-level benefit by precluding protection of dengue naïve individuals against DENV1 and DENV2 and complicating vaccine delivery.
• The concern was raised that potential serotype replacement with DENV3 and/or DENV4 could lower vaccine impact and potentially increase the risk of DENV3 and DENV 4 disease among dengue naïve vaccinees over time. IVIR-AC recommends a systematic examination of whether the models predict increased circulation of DENV3 and/or DENV4 following vaccination.
• Both models suggested that the population-level impact of TAK-003 would be greater if vaccination prevents infection and not just disease. However, serotype replacement may be more likely if vaccine protection is primarily against infection rather than disease. Further studies are needed to better understand the nature of vaccine protection against all serotypes and the impact of TAK-003 vaccine on transmission.

Additional recommendations include:

• IVIR-AC recommends highlighting that models incorporated all available trial data to best inform vaccine parameters. Nevertheless, data limitations exist around DENV3 and DENV4 endpoints, and attack rates were lower in years 4 and 5 (and there is no follow-up beyond year 5), which limited the ability to estimate longer-term waning of vaccine protection. Moreover, models differed in vaccine-related assumptions (i.e., time-varying serotype-specific vaccine efficacy). Much care is needed when communicating the uncertainties regarding model results.
• Similarly, in the TAK-003 vaccine trial, hospitalization was used as a marker for severe disease, but hospitalization practices varied substantially across trial sites in various countries which reflect local standards of medical practice and diagnostic resources. Additionally, the modelling groups made different assumptions regarding the probability of hospitalization. Therefore, the results for the model-predicted hospitalizations should be interpreted with caution.
• For the Imperial College London group:
  o IVIR-AC notes that it would be useful to further clarify how antibody dynamics were used to model waning of vaccine protection and the risk of negative vaccine efficacy against certain serotypes. IVIR-AC recommends considering the implications for extrapolating estimates of vaccine efficacy beyond the follow-up period of the trials.
  o IVIR-AC recommends comparing results between TAK-003 and Dengvaxia with their revised model and further examining the robustness of evidence for vaccine induced risk enhancement of DENV3 and DENV4.
• For the University of Notre Dame group:
  o IVIR-AC notes that it might be more valuable to model “country-archetypes” (e.g., one city or country per WHO-affected region) and recommends including the range or credibility intervals to reflect the uncertainties in parameters values.
  o Age of vaccination and coverage assumptions need to be more clearly stated. IVIR-AC also recommends including age structure in the model, which would simplify how age-specific vaccination strategies were modelled.
  o This model is unlikely to be able to capture vaccine impact on disease transmission in the presence of differential serotype-specific vaccine efficacy and the potential for serotype replacement since it does not explicitly model the individual serotypes. This could lead to overestimation of the longer-term impact of vaccination.
  o IVIR-AC commends the group on the comparison between TAK-003 and Dengvaxia, which provides useful context.
• In view of the above and their implications for vaccine confidence, IVIR-AC notes that it will be important to formulate and pre-test clear communication around uncertainties in the individual-level benefits and risks.

### 3.5 Session 5: Mathematical modelling of the COVID-19 pandemic according to different vaccination scenarios in Burkina Faso and Cameroon

Since the emergence of SARS-CoV-2, mathematical modelling has served as an important tool to support decision-making. While many high-income countries benefited from mathematical model projections to guide public health measures, these models remain mostly inaccessible to decision-makers in low- and middle-income countries (LMICs). Thus, mathematical models and their subsequent results often have sub-optimal use to inform public health decisions in these settings. Currently, there is limited capacity to generate and use modelling in the WHO African region and there were few experiences of mathematical modelling in the African region during the COVID-19 pandemic that involved local modellers. Therefore, there is a need to build local capacity in modelling throughout the region, as well as a need to prepare for a resurgence of COVID-19 or emergence of a future pandemic.

In response, the WHO African Regional Office (AFRO) and the research group in mathematical modelling of the Centre de Recherche du CHU de Québec (Université Laval, Canada) started a project in 2022 to build COVID-19 modelling capacity in French and English-speaking African countries. The main objective of the project was to develop fit-for-purpose, country-specific, mathematical models of COVID-19 with local researchers that could be used to predict the trajectory of the COVID-19 pandemic according to various scenarios. Specific aims of this project include:

• To identify 2–4 interested countries, with available data and capacity to participate;
• To establish a collaboration with country-specific researchers and decision makers to identify the data and modelling needs and aims;
• To develop and calibrate an open-source dynamic mathematical model of COVID-19 transmission, in close consultation with countries through regular meetings and an in-person workshop;
• To examine the potential evolution of the COVID-19 pandemic according to the different vaccination scenarios;
• To hold a close-out simulation workshop to present the modelling methods and interface and to review different steps of model simulation using applied case-based scenarios; and
• To publish at least one manuscript with participating countries.

This project was implemented on the principle to foster a partnership between an institution with modelling specialty (Université Laval) and local research groups and is currently testing a pilot of this approach. Following meetings organized by WHO, two countries were chosen to participate based on interest and capacity to engage: Burkina Faso and Cameroon. During the session, WHO AFRO presented an overview of the general concept and overall project objectives. The Université Laval team then presented the specific aims of the project and progress to date. Since late 2022, the Université Laval team has held ongoing meetings, some of which were in-country, with the teams in Burkina Faso and Cameroon to discuss the aims of the project, the context of COVID-19 within each country, possible model structures and sources of data, and to discuss future steps.

The Université Laval team developed a dynamic compartmental model of COVID-19 implemented in Python that includes data on demographic information and contact patterns along with the ability to account for vaccination and variants of concern. Outputs of the model are estimates of detected cases, resource use (e.g., clinic visits, hospitalizations), and deaths. Models in both countries are currently being calibrated to normalized COVID-19 incidence data through an iterative fitting algorithm that generates updated parameter sets by combining prior tested parameter sets. The Université Laval team is working with local research teams to develop various scenarios for testing, including with regards to vaccination and variants of concern. In December 2023, the team will hold a close-out meeting to present the final modelling methods and interface, use the simulation framework to generate results under different scenarios, and get overall feedback on the model interface and documentation.

IVIR-AC was asked to provide feedback on the project methods and proposed modes of engagement with country collaborators, researchers, and decision makers for the close-out simulation workshop.

IVIR-AC feedback and recommendations

IVIR-AC recommended that as other health priorities take precedence over COVID-19, common modelling interests beyond COVID-19 should be explored at the close-out meeting as a means to sustain or further develop capacity that was built and ensure continuity in engagement in modelling. These recommendations include:

• The close-out meeting should incorporate participants’ reflection on the process and outcomes of the project and evaluate perceptions of whether the process has enhanced modelling capacity and/or the ability to interpret and communicate outputs from models.
• The scope of potential model outputs should be clearly defined, in terms of the types of questions the model is able to answer, and those beyond the scope.
• Model documentation should be prioritised. Tools such as user guides with screenshots, a database of parameter and data sources, video demonstrations, and options for version control and in-house model updates could be implemented.
• To support the use of the user-friendly modelling tool, users should be provided with clearly defined questions to model beyond the close-out meeting.

Additional recommendations include:

• The suitability of the models for producing short-term predictions versus long-term scenarios should be considered and clearly communicated with country groups.
• For the model to remain relevant, updates to the structure, assumptions, parameters, scenarios, and outputs produced may be needed. IVIR-AC recommends considering who would have responsibility and capacity for making these types of updates.
• IVIR-AC recommends a pilot simulation exercise could be implemented in advance of the close-out workshop to highlight areas for improvement and clarification.
• IVIR-AC notes that future efforts should focus on different levels of capacity building and modelling needed including using and interpreting model outputs, understanding how models work, adapting existing models, and constructing original models.

### 3.6 Session 6: Therapeutic HPV vaccine impact modelling

In 2020, WHO launched a global strategy to eliminate cervical cancer with targets [14] (hereafter 90-70-90 targets) to achieve 90 % coverage with preventative vaccination among girls by age 15 years, 70 % of women screened for cervical disease with high performance tests by 35 years of age and again by 45 years of age, and 90 % of women identified with cervical disease receive treatment by 2030. If these targets are met and sustained, over 62 million deaths could be averted over the next 100 years. To reach these ambitious targets, there needs to be scale up of existing interventions but also exploration of new innovations that might enhance existing efforts or address specific gaps.

Development of a therapeutic human papillomavirus (HPV) vaccine may provide an important addition to the current methods to prevent and treat cervical cancer, especially among women in LMICs, who often lack access to screening and treatment. Therapeutic HPV vaccines, which are designed to clear or treat existing HPV infections or HPV-associated cervical lesions rather than prevent infection, are currently in early clinical development (with several candidates in phase I/II trials) and might offer an additional tool to address the gaps in current cervical cancer programmes. A WHO-convened group of experts identified two overarching contexts with public health needs for therapeutic HPV vaccines:

• In settings where it is difficult to scale up cervical cancer screening and treatment, reaching women who have been infected and likely had not received preventative HPV vaccines would reduce the overall proportion that develop cervical precancers, and
• In settings where some screening and treatment has been implemented (which however is costly, complex, or has large loss-to-follow-up), providing an alternative, simpler treatment following a positive test for HPV infection, would increase the overall proportion of women with precancers who are effectively treated.

Draft PPCs for therapeutic HPV vaccines have since been developed and posted for public comment [15], which address the following two therapeutic vaccine approaches:

• Vaccines that clear oncogenic HPV infection to be used in adult women through population-based vaccine delivery without a preceding HPV screening test.
• Vaccines that cause regression of high-grade cervical precancers to be used mainly as an alternative or adjunct to existing cervical precancer treatments following a positive HPV screening test.

A team from The Daffodil Centre at the University of Sydney (A Joint Venture with Cancer Council NSW, Australia) presented results from a modelling analysis on therapeutic HPV vaccine impact on cervical cancer incidence and mortality and cost effectiveness in LMICs across various use cases and preferred vaccine characteristics. This modelling process was informed by an expert advisory group and multiple workshops on specific topics (e.g., vaccination development timelines, vaccine efficacy, potential use of a therapeutic vaccine, considerations for women living with HIV). Three main Use Cases were defined and mapped to PPCs and vaccine efficacy levels: 1) population-level untargeted mass vaccination of all adult females in an age cohort, 2) targeted therapeutic vaccination within a screen-and-treat programme, only offering therapeutic vaccine to screen-positive women, and 3) test-and-vaccinate with therapeutic vaccine, outside screen-and-treat programmes, only offering therapeutic vaccine to HPV-positive women. Use Cases 1 and 3 were mapped to a PPC where the vaccine acts primarily against infection and has an efficacy against high grade cervical lesions of 50 to 90 %. Use Case 2 was mapped to a PPC with a vaccine that acts against high-grade cervical lesions with an efficacy ranging between 0 % and 90 %.

The modelling team used the Policy1-Cervix platform [16], which has previously been used for WHO elimination modelling exercises and to support updated WHO screen-and-treat guidelines. The model leverages natural disease history transitions while overlaying possible interactions with a screening programme and is calibrated to incidence and mortality data from 78 LMICs. Modelling simulations tested various preventative vaccination coverage, screening and treatment levels. The model was updated to simulate both the impact of implementing a therapeutic vaccine that acts to increase infection clearance and another to increase cervical lesion regression. The team also conducted a cost-effectiveness analysis under various Use Cases and PPCs.

IVIR-AC reviewed information provided during the session, and given the timelines of emerging information, a full evaluation was not possible. IVIR-AC provided the following comments based on discussion during the meeting:

• IVIR-AC discussed the need to consider the acceptability, feasibility, ethical and regulatory implications of mass therapeutic vaccination in the absence of screening.
• IVIR-AC affirmed the need for a clear communication strategy to differentiate between preventative and therapeutic vaccines.
• IVIR-AC acknowledged modelling the therapeutic vaccine against a baseline of no further scale-up towards the WHO 90-70-90 targets, and discussed the need for comparison to realistic scale-up levels in addition to considering the future value of the therapeutic vaccine after the achievement of the WHO 90-70-90 targets.

### 3.7 Session 7: Vaccine Impact Modelling Consortium

The Vaccine Impact Modelling Consortium (VIMC) [17] is a multinational collaboration, composed of 21 research groups funded by Gavi, the Vaccine Alliance, the Wellcome Trust, and the Bill & Melinda Gates Foundation (BMGF). VIMC includes modelling for 12 different diseases including: cholera, hepatitis B, *haemophilus influenzae* type b, HPV, Japanese encephalitis, meningitis type A, measles, pneumococcal disease, rotavirus, rubella, typhoid, yellow fever, COVID-19, and malaria. The VIMC began its second five-year grant phase (VIMC 2.0) in September 2022, which will run through 2027, with a focus on responsive policy-driven mathematical modelling to answer priority research questions and the following core aims:

(1) to provide reliable and accessible estimates of vaccine impact across the Gavi portfolio;
(2) to address critical modelling-related vaccine policy questions raised by stakeholders who will be dynamically engaged in the work;
(3) to translate the Consortium’s modelling to real-world policy that improves health outcomes;
(4) to foster a diverse international community of vaccine impact modellers, inclusive of modellers in low- and middle-income countries (LMICs); and
(5) to provide training in infectious disease modelling and its application to vaccine-preventable diseases for both modellers and policy makers.

VIMC additionally established a new research agenda to investigate the impact of climate change on vaccine-preventable diseases sensitive to climate. In the new grant phase, VIMC re-established engagement with WHO IVB through a focused collaboration under the WHO Collaborating Centre for Infectious Disease Modelling agreement and have focused collaborative efforts on Impact Goal indicator 1.1 (“number of future deaths averted through immunization”) for IA2030 vaccine impact estimates (see next session for more information), which specifically include mid-point target updates for IA2030 vaccine impact estimates by 2025 and modelling analyses to answer priority questions raised by IA2030 partners and WHO IVB.

To address aims 2 and 3 above, VIMC established modeller-led project working groups (PWGs) with the objective to answer discrete, policy-relevant questions in collaboration with a diverse range of engaged stakeholders and then to translate and disseminate results with the ultimate objective of creating real-world health benefits. During the session presentation, VIMC presented the process for defining research questions and initiating PWGs within the Consortium, including contact information for posing a question for consideration (vimcquestions@imperial.ac.uk). The presentation highlighted ongoing and completed PWG projects including to assist in decision making to set elimination targets for measles and rubella in the WHO South-East Asia Region (see further information on specific session), to inform cholera outbreak response guidelines, to optimise measles SIAs as well as timing of yellow fever outbreak response and schedules for the introduction of meningococcal vaccines. Four of ten eligible core disease areas are currently engaged in PWGs and stakeholders to date have included WHO, CDC, Unicef, Gavi, and BMGF.

VIMC also presented an update on plans and actions for the next round of full model estimates, where each modelling group runs a range of vaccination scenarios and impact is calculated by the central Secretariat. This will only be one full model run in VIMC 2.0 and will be completed by mid-2024. Full model runs by disease will include four different coverage scenarios: where no vaccination exists, the “default” or current vaccination coverage levels reached historically and projected to occur if business remains as usual, coverage meets IA2030 targets, and an even more optimistic “blue-sky” targets (e.g., 90 to 95 % coverage). Implementations of future SIA events for projections are informed by WHO guidelines and planned activities under each scenario.

IVIR-AC was asked to review and provide feedback on the PWG strategy and progress to date, the process to identify future priority questions for PWGs, and to highlight considerations for communicating future VIMC full model estimates in 2024.

IVIR-AC feedback and recommendations

Overall, IVIR-AC recognizes the value of evidence generated by the PWGs and largely agrees with the workflow of the PWG to address relevant policy questions. IVIR-AC highlighted the importance of ongoing model validation for each pathogen against historical data for the upcoming full model runs and recommends the following:

• To gain the confidence of non-modellers in the model results, it would be useful to validate the models with historical input data to determine how well they predicted what actually occurred with a given pathogen and vaccination strategy.
• For the PWGs:
  o VIMC should clarify the diversity of stakeholders, including partners and immunization programme managers, and the processes for identifying policy questions for the PWGs that can be addressed or informed by using modelling approaches.
  o IVIR-AC recommends regular communication with the Strategic Advisory Group of Experts on Immunization (SAGE) Secretariat, working group focal points, and Gavi Vaccine Investment Strategy (VIS), as those are key sources to identify short term questions that the PWGs are equipped to address.
  o The VIMC Secretariat should provide flexible funding, beyond the scope of ongoing commitments as part of the funding through VIMC, to members who are asked to participate in multiple PWGs, and governance mechanisms must ensure the independence of the PWGs from stakeholder incentives.
  o VIMC should conduct a more in-depth review of the progress made by PWGs in answering critical questions, such as: how many policy questions were proposed to the PWG, by whom; reasons for decision to address or not address a specific policy question; reasons for success and timely completion and failure of the task; and whether the outputs had the desired impact. For example, it should be investigated if the PWG outputs have been shared with countries and if there is any feedback from the countries or on this process.
  o VIMC should identify the two research groups responsible for modelling each pathogen and the level of preparedness to engage in new projects in view of available resources and ongoing projects. Also, IVIR-AC recommends clarifying procedures for incorporating new pathogens into the VIMC portfolio.
  o Possible questions for PWGs to consider could include the following:
    ▪ What is an effective combination of vaccines that can be given in a campaign in a humanitarian crisis?
    ▪ What is the likely impact of maternal RSV vaccine in VIMC countries?
• For the upcoming full model runs, IVIR-AC recommends:
  o developing a communication plan for model dissemination;
  o identifying the synergies and interdependencies with IA2030 and other related work;
  o providing code and parameters for each model for transparency and reproducibility; and
  o indicating the level and major drivers of uncertainties in the vaccine impact estimates for each pathogen.
• Finally, VIMC should generally continue to identify the synergies and interdependencies with IA2030 and other related work packages.

### 3.8 Session 8: Immunization Agenda 2030 vaccine impact estimates

The Immunization Agenda 2030 (IA2030) vaccine impact estimates project has the following objectives:

• To estimate the impact of vaccination by achieving the ambitious goals set out for IA2030,
• To inform strategic priorities for the IA2030 and Triple Billion targets for WHO’s Thirteenth General Programme of Work (GPW13), and
• To provide an indicator for the IA2030 Monitoring & Evaluation framework [18], which has been set as Impact Goal indicator 1.1 and is the “number of future deaths averted through immunization” for WHO’s 194 Member States from 2021 to 2030.

The project has completed the first round of target setting [19] for 14 pathogens to include mortality impact and routine immunization data and leveraged existing VIMC estimates and input from Gavi and the IA2030 Stakeholder Committee. The first round of the project estimated that vaccinations administered from 2021 to 2030 would avert approximately 51.5 million deaths (95 % Confidence Interval: 44.0 – 63.2 million deaths), most of which were attributed to measles and hepatitis B and across low- and lower-middle-income countries. By 2025, the project plans to update target estimates by adding more pathogens, incorporating Disability-Adjusted Life Years (DALYs) with target metrics, adding coverage data from SIAs, updating methods to include a validation framework, consulting with disease expert groups, and focusing on documentation and reproducibility. The next project phase through 2025 will also align with VIMC 2.0 and Gavi 6.0, and following this phase, the project will move to an annual progress tracking cycle where annual IA2030 technical progress reports will be presented to SAGE and the IA2030 scorecard [20] will be updated each year following the WUENIC coverage release. Since the project’s inception in 2020, IVIR-AC has provided independent reviews of models, analytical frameworks, and methods to inform the use of estimates for global and regional policy and planning process. Following earlier recommendations, IVIR-AC previously reviewed the results of an uncertainty analysis and provided feedback on approaches to annual monitoring and reporting, especially regarding communication strategies.

During the session, the project team reviewed the process of target setting which includes using non-linear scaling of WUENIC coverage estimates, of the third dose of diphtheria-tetanus-pertussis vaccine (DTP3) in 2030, accounting for the COVID-19 pandemic, as country-specific endpoints. Vaccine introductions are assumed to be evenly dispersed from 2023 to 2027 such that countries with higher DTP3 coverage endpoints have earlier introductions. Target vaccine coverage is then converted to target deaths averted by first converting coverage percentages into fully vaccinated persons (FVPs) by using population forecasts; then, FVPs are converted to deaths averted using an impact factor that summarizes the number of deaths averted over a lifetime per FVP. The project team highlighted that as WUENIC revises historical DTP3 coverage estimates with new releases and DTP3 2030 coverage targets change, population forecasts are updated yielding different estimates. The team proposed changing targets with each annual IA2030 impact estimate update to align with the current observed data. This approach at the country-level yields some notable changes for disease-specific baseline values per country, but at a global scale does not yield significant variation in final target metrics.

Including DALYs as target metrics in the IA2030 impact framework would allow for a more complete understanding of the ability of vaccination to reduce disease burden. DALYs are already estimated for diseases modelled by VIMC, but introducing estimates of DALYs for diseases outside the VIMC framework requires new methods which are currently under development by the project team. The project team presented their proposed approach which includes the following:

(1) First, back calculating incidence using CFR and estimates of deaths under a vaccination and no-vaccination scenario.
(2) Next, defining disease states as described by the Global Burden of Disease study, and for diseases with multiple states, use morbidity rates to estimate the proportions of new cases developing sequalae.
(3) Then, calculating years of life with disability (YLD), using the number of cases, the number of years expected to be in each disease state, and the disability weights associated with each disease state.
(4) Next, calculating years of life lost (YLL) using the number of deaths and the difference between life expectancy and age at death.
(5) Finally, computing DALYs by combining estimates of YLDs and YLLs for both vaccine scenarios, and taking the difference in DALYs under both scenarios to compute DALYs averted.

To include non-routine vaccination coverage (e.g., from SIAs), the project team overviewed the scope of activities that would need to be considered in the estimation framework, highlighting most SIAs occur to prevent measles and polio and that a majority of SIAs occur in VIMC countries. The impact per campaign however is not consistent, which will likely pose challenges to using an impact factor-based approach for target metric computation. Alternatively, the project team presented on plans for considerations of a ‘non-linear’ relationship between FVPs and deaths averted, which includes fitting multiple models to curves of cumulative FVPs versus cumulative deaths averted that represent different assumptions of impact and select models that best fit empirical data. The project team concluded by briefly sharing plans to update uncertainty metrics to be from triangular distribution instead of beta distribution for non-VIMC diseases to better match empirical data and by outlining details regarding an open-source R package [21] with the objective of sharing analysis code with the broader immunization research community.

IVIR-AC was asked to provide feedback on the team’s ongoing efforts to update the target estimates by 2025, specifically on communicating progress towards Impact Goal 1.1, estimating DALYs, and the suitability of an open-source R package.

IVIR-AC feedback and recommendations

Beyond acknowledging the response to previous recommendations and revised methods, IVIR-AC recommends clarifying the terminology used and the online dissemination strategy of the project code base, including the following:

• Some of the terms used, such as “future deaths” or “observed deaths averted”, could be liable to misinterpretation. IVIR-AC suggests clearly defining various terms and using them consistently.
• Regarding the inclusion of DALYs in target metrics, IVIR-AC finds the proposed approach sound, and encourages systematic identification of key drivers of heterogeneity in case fatality and basing the stratification of estimates on identified drivers.
• IVIR-AC notes that the uncertainty in estimates of both deaths and case fatality ratios need to be propagated in the estimates of DALYs. This could be done via bootstrapping methods.
• IVIR-AC commends the open-source sharing of project code, recommends additional documentation and communication on use of the code, and advises considering the merit of developing a supplementary online tool or dashboard to allow user-friendly data exploration.
  o IVIR-AC emphasised the need for clear communication and documentation of any online tool or resource to minimise the risk for misinterpretation

Additional recommendations include:

• IVIR-AC recommends adding clarity that other potentially impactful vaccines (e.g., respiratory syncytial virus (RSV), malaria) are not included in these estimates.
• IVIR-AC notes that data sharing plans are not the same as science communication strategies. When communicating the estimates, one shall consider existing evidence on communicating uncertainty which recommends, for example, forewarning audiences about the limitations of the estimates, using lexical hedges (e.g., “approximately”) and ranges in estimates (e.g., is estimated to be *x* but could be as low as *a* and as high as *b*) [22].
• IVIR-AC recognizes that analyses for this project will hinge on robustness and availability of data for parameterisation (e.g., CFRs, vaccine efficacy, disability weights). The project team will need to clearly document limitations and their related implications for the estimates.
• IVIR-AC commends the figure highlighting differences between achieved impact versus anticipated impact as a useful addition and recommends that it would be worth highlighting regional differences by using a standardised y-axis.

## 4 Recurring themes

Across sessions, recommendations and discussion points consistently emphasised the following that (1) models across sessions and disease areas need to be validated against historical data as an essential step to gain trust of non-modellers and policymakers, ultimately strengthening the impact on programmatic decision-making; (2) area-specific immunization context and barriers to implementation, including feasibility, acceptability and regulatory issues, need to be well characterised before modelling the impact of vaccination; (3) modelled results and uncertainties, as well as assumptions made by and limitations inherent to different modelling methods, need to be clearly and adequately communicated to users with varying technical levels; and (4) that translating modelling recommendations to actionable policy evidence and implementation requires sustained capacity building including training on both developing new models as well as interpreting models and modelled evidence.

IVIR-AC will next convene virtually from 26 February – 1 March 2024 to discuss an agenda that will be soon determined.

## CRediT authorship contribution statement

**Philipp Lambach:** Conceptualization, Project administration, Supervision, Writing – review & editing. **Walt Orenstein:** Investigation, Supervision, Writing – review & editing. **Sheetal Silal:** Investigation, Supervision, Writing – review & editing. **Alyssa N. Sbarra:** Conceptualization, Writing – original draft. **Mitsuki Koh:** Project administration, Writing – review & editing. **Rakesh Aggarwal:** Investigation, Writing – review & editing. **Habib Hasan Farooqui:** . **Stefan Flasche:** Investigation, Writing – review & editing. **Alexandra Hogan:** Investigation, Writing – review & editing. **Sun-Young Kim:** Investigation, Writing – review & editing. **Julie Leask:** Investigation, Writing – review & editing. **Paula M. Luz:** Investigation, Writing – review & editing. **Dafrossa C. Lyimo:** Investigation, Writing – review & editing. **William J. Moss:** Investigation, Writing – review & editing. **Virginia E. Pitzer:** Investigation, Writing – review & editing. **Xian-Yi Wang:** Investigation, Writing – review & editing. **Joseph Wu:** Investigation, Writing – review & editing.

## Declaration of competing interest

The authors declare that they have no known competing financial interests or personal relationships that could have appeared to influence the work reported in this paper.

## Data Availability

No data was used for the research described in the article.
